# Supplementary material for: Investigating a visceral measure of perceived physical attractiveness
Source: PLoS One. 2024 Nov 21;19(11):e0311138. doi: 10.1371/journal.pone.0311138 (PMC11581254; doi:10.1371/journal.pone.0311138)
Supplement: Attachment — Submitted filename: Response to Reviewers.docx [file pone.0311138.s001.docx]

# **Jayaprakash Narayana Kolla** August 15, 2024

**Academic Editor**

# **PLOS ONE**

Dear Dr. **Kolla**,

We very much appreciate your invitation to revise and resubmit for publication consideration our manuscript entitled, “Investigating a Visceral Measure of Perceived Physical Attractiveness” [PONE-D-24-24619]. This letter details our point-by-point responses to the comments provided by the reviewers, who offered valuable recommendations for our manuscript.

**Journal Requirements**

1. *Please ensure that your manuscript meets PLOS ONE's style requirements.*

We have addressed the style requirements.

2. *Please expand the acronym “NHLBI” (as indicated in your financial disclosure) so that it states the name of your funders in full. This information should be included in your cover letter.*

We have expanded the acronym in the financial disclosure and now note funding in the cover letter.

3. *Please include your full ethics statement in the ‘Methods’ section of your manuscript file. In your statement, please include the full name of the IRB or ethics committee who approved or waived your study, as well as whether or not you obtained informed written or verbal consent. If consent was waived for your study, please include this information in your statement as well.*

This information is included in the methods section (p.4): “This report derives from a study approved by the University of Pittsburgh’s IRB. During this two-session study, participants provided written informed consent…”

**Reviewer 1**

1. *In the methods, the authors state that the alcohol component of the study does not play a role in this report. But a few lines further down, they say that a PPA task was completed after the second drinking period. This sounds like a contradiction to me. Please explain.*

While the larger study was focused on drink condition differences, the goal of the task that is the focus of the present report was to compare two measures of attractiveness perceptions (dynamometer and verbal) irrespective of drink condition. As noted on lines 95-96, we control for drink condition in all analyses and it was not significant. We do not elaborate further on the drink condition, but instead refer readers to other publications for details on the drink manipulation if of interest (line 68).

2. *The images to assess included 4 male and 4 females. This looks like not enough to get a relevant result. Please explain.*

This is a within person comparison of two attractiveness measures (dynamometer and verbal). To control for the impact of target on measure variance, both measures are based on the same set of 16 facial images (8 targets). There is not reason to think more targets would alter the findings, as they would be presented in both assessment modes.

3. *How is the squeezing standardized? I assume, different people have different squeezing strength but want to express a similar thing. Whereas the same strength might mean something different to different individuals. Base on this assumption, I also can not see how squeezing strength and verbal expression can be associated.*

As noted above, this is a within person comparison. We assessed the relation between squeezing and verbal ratings within each target, within each participant. Thus, each participant serves as their own control. The random intercept in the mixed effects models accounted for potential variation across participants.

**Reviewer 2**

We were please Reviewer 2 found our paper to report on “important research in social psychology” that will “further the study of interpersonal attraction.” This reviewer had no requested revisions.

In closing, it is our hope that we have adequately addressed the recommendations offered to us and we look forward to hearing from you.

# Sincerely,

Molly A. Bowdring, PhD
